# Recognizing acute promyelocytic leukemia in a patient with treated chronic lymphocytic leukemia

**DOI:** 10.1002/jha2.138

**Published:** 2020-11-25

**Authors:** Suparna Nanua, Furha Cossor, Shahzad Raza

**Affiliations:** ^1^ Saint Luke's Hospital of Kansas City Kansas City Missouri; ^2^ Saint Luke's Cancer Institute University of Missouri Kansas City Kansas City Missouri; ^3^ Department of Internal Medicine, Division of Hematology Oncology University of Missouri Kansas City Kansas City Missouri

An 83‐year‐old gentleman with history of chronic lymphocytic leukemia (CLL) characterized by multiple cytogenetic abnormalities (trisomy 12, loss of 13q14.2 and 13q34, and loss of *TP53*) received prior chemotherapy (CHOP‐R and BR) 4 years ago presented with pancytopenia (white blood cells 1.2 × 10^9^/L, hemoglobin 72 g/l, platelet count 30 × 10^9^/L) while on ibrutinib 420 mg orally daily. Flow cytometric analysis from bone marrow biopsy showed two distinct population‐ first, bright CD45+ lymphocytes (red), consisting of clonal B cells co‐expressing CD20+/CD5+ (Figure 1: A‐C), and, second, dim/moderate CD45+ (green), negative for CD34 and HLA‐DR expression, and positive myeloid markers CD13, CD33, and CD117, suspicious for atypical promyelocytes (Figure 1: D‐F). Bone marrow aspirate smears showed residual CLL (Figure 1:G‐H; X 500[G] and x 400[H] Wright‐Giemsa stain) and concurrent atypical promyelocytes including “faggot cells” (Figure 1: G‐H). Circulating neoplastic cells were identified in peripheral blood (Figure 1: I; H&E stain X 100). Bone marrow core biopsies similarly revealed lymphoid aggregates consisting of mostly small B cells admixed with atypical promyelocytes (Figure 1: J; H&E stain X 100). Fluorescence in situ hybridization showed a *PML/RARA* rearrangement in 44% of interphase. Patient was initially treated with all‐trans retinoic acid and arsenic trioxide induction regimen for 3 weeks. However, he declined further systemic treatment and decided comfort care.

Acute promyelocytic leukemia is uncommon in CLL and is mainly associated with prior exposure to topoisomerase II inhibitors and ionizing radiation. Prolong immunosuppressions either secondary to chemotherapy or CLL with complex karyotype, *per se*, are possible causes of secondary myeloid malignancy. Oncologist should be aware of concurrent and/or evolving hematologic malignancies in CLL patients.

**FIGURE 1:**
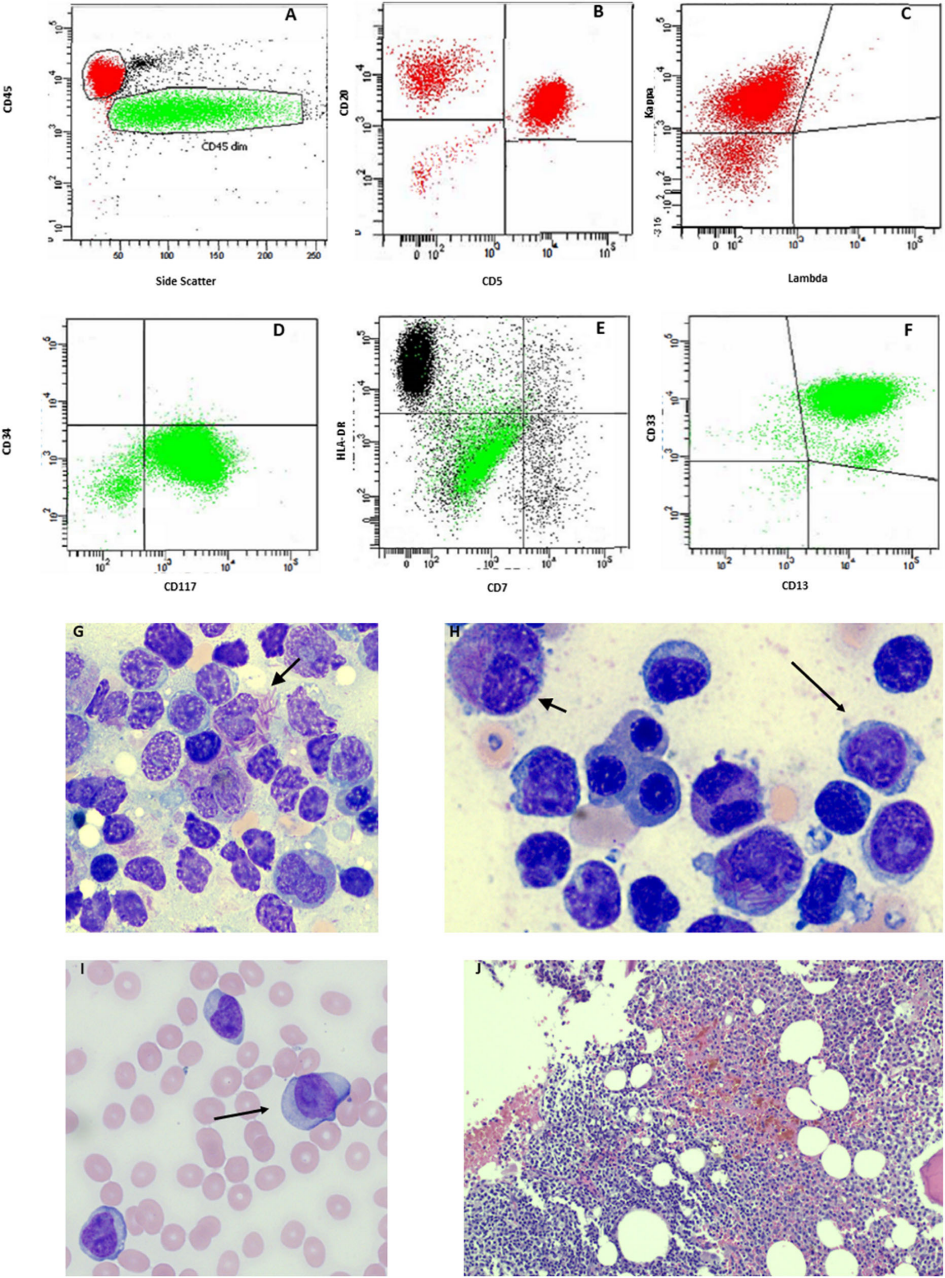
Flow cytometric analysis of lymphocytic and blast population. Bright CD45 positive lymphocytes (red), co‐expressing CD20 and CD5, with Kappa surface light chain restriction (Panel A‐C). Dim/moderate CD45 positive cells (green), negative for CD34 and HLA‐DR expression, and positive myeloid markers CD13, CD33, and CD117 (Panel D‐F). Chronic lymphocytic leukemia lymphocytes (arrow) in bone marrow aspirate and peripheral blood smears and abnormal promyelocytes with numerous auer rods (arrow head) in bone marrow aspirate smears (Panel G‐H). Circulating neoplastic cells were identified in peripheral blood (Panel I). Bone marrow core biopsies similarly revealed lymphoid aggregates consisting of mostly small B cells admixed with atypical promyelocytes (Panel J).

## CONFLICT OF INTEREST

Suparna Nanua and Furha Cossor have no conflict of interest, and Shahzad Raza received honorarium in participation of advisory board of Amgen, Janssen, Celgene, Takeda, Incyte, Novartis, and Takeda, and he is also Speaker Bureau for Janssen, Takeda, and Celgene.

